# Regional Dependence of Corvis ST Biomechanical Parameters on Corneal Material Properties: A Finite Element Study

**DOI:** 10.3390/bioengineering13080904

**Published:** 2026-08-10

**Authors:** Zeyu Li, Yixin Zhang, Yijian Wang, Yaohua Liu, Xuefei Li, Xue Zhai, Yudi Han, Ahmed Elsheikh, Shenglong Luo, Junjie Wang

**Affiliations:** 1 National Engineering Research Center of Ophthalmology and Optometry, Eye Hospital, Wenzhou Medical University, Wenzhou 325027, China; 2 State Key Laboratory of Eye Health, Eye Hospital, Wenzhou Medical University, Wenzhou 325027, China; 3 Oujiang Laboratory (Zhejiang Lab for Regenerative Medicine, Vision and Brain Health), Eye Hospital, Wenzhou Medical University, Wenzhou 325027, China; 4 School of Engineering, University of Liverpool, Liverpool L69 3GH, UK

**Keywords:** ocular biomechanics, finite element analysis, parametric study, Corvis ST

## Abstract

Background: Corvis ST (CVS) provides dynamic corneal response parameters for biomechanical assessment, but conventional metrics may not uniquely reflect region-specific alterations in corneal material properties. This study investigated how regional changes in corneal material properties influence CVS biomechanical parameters and explored a multi-parameter strategy for regional mechanical interpretation. Methods: An idealized finite element corneal model was divided into central, paracentral, and peripheral zones, and the stiffness of each zone was independently adjusted to 70–130% of baseline to simulate regional softening or stiffening. An air-puff load was applied to the anterior corneal surface, and nodal coordinates were used to calculate conventional CVS parameters and the newly proposed DA Ratio X series. Normalized relative changes and univariate linear regression were used to characterize response direction and sensitivity. Results: Most parameters showed approximately linear responses to regional stiffness alterations, with region-dependent differences in response direction and magnitude. Central material changes produced the largest responses. Similar parameter values or trends could arise from different regional mechanical alterations, indicating that a single parameter may not uniquely identify the underlying regional change. Within this idealized simulation framework, the extended DA Ratio series improved regional differentiation, and the combination of DeflArea, DAR4, and IIR provided a candidate theoretical framework for distinguishing central, paracentral, and peripheral changes. Conclusions: CVS biomechanical parameters were influenced by the spatial location of corneal material alterations, and single-parameter interpretation may not uniquely reflect the underlying regional mechanical changes. Coordinated interpretation of multiple parameters demonstrated distinct response patterns across different modeled regions and may provide ideas for future regional biomechanical assessment. Further validation using patient-specific models and clinical datasets is warranted to evaluate the applicability of this framework.

## 1. Introduction

As the primary refractive structure of the human eye, the cornea accounts for approximately two-thirds of the eye’s total refractive power, and its morphological and biomechanical properties directly determine visual quality [1,2]. Mounting evidence indicates that corneal biomechanical alterations often precede morphological abnormalities and may serve as the initiating factor leading to the deterioration of corneal morphology [3,4]. Corneal biomechanical assessment has been widely applied in early screening of keratoconus (KC) and safety evaluation of laser vision correction (LVC) surgery [5,6].

Corneal Visualization Scheimpflug Technology (Corvis ST, CVS) is a non-contact tonometer based on ultra-high-speed Scheimpflug imaging that captures real-time dynamic deformation of the corneal cross-section along the horizontal meridian [7,8]. A series of quantitative parameters, collectively referred to as dynamic corneal response (DCR) parameters, are extracted from these imaging data [9,10]. Compared with the Ocular Response Analyzer (ORA), CVS substantially enhances the dimensionality and accuracy of in vivo corneal biomechanical evaluation by temporally analyzing mechanical characteristics at each deformation stage [11,12,13], thereby providing more robust evidence for KC diagnosis and refractive surgery risk assessment [14,15].

Meanwhile, accumulating evidence indicates that conventional CVS parameters are insufficient for regional differentiation of corneal biomechanics [16]. These parameters are derived under the assumption of a homogeneous corneal material, whereas the native cornea is a nonlinear, anisotropic biological composite with marked regional variations in collagen fiber architecture and mechanical properties [17,18]. Notably, clinical observations have revealed inconsistent and paradoxical postoperative parameter changes: 3–6 months following refractive surgery, DAR2 decreased (suggesting increased stiffness) while IIR simultaneously increased (suggesting decreased stiffness) [19]. Similarly, intracorneal ring segment (ICRS) implantation in keratoconic eyes induced no significant changes in any standard CVS parameters, despite its well-documented mechanical reinforcement effect [20]. These findings collectively reveal that fundamental yet unexplored questions remain: can CVS parameters reliably reflect biomechanical changes across different corneal regions, and does a given parameter change correspond uniquely to a specific underlying mechanical alteration? These questions carry significant clinical implications.

Current methods for assessing corneal biomechanics include ex vivo experiments, in vivo measurements, and finite element (FE) simulations. Ex vivo animal corneal tests provide detailed stress–strain data but cannot fully replicate human physiology due to species differences, absence of intraocular pressure, and tissue fixation artifacts. In contrast, FE simulations allow controlled variation for model inputs such as corneal geometry and surgical parameters, and systematic comparison of corneal biomechanical responses [21,22], while recent advances in material characterization and generalized mechanical formulations are improving the physiological realism and predictive capability of corneal FE models [23,24].

This study aimed to investigate the effects of regional alterations in corneal material properties on CVS biomechanical parameters using finite element analysis (FEA), clarify the scientific implications of CVS parameters, and provide theoretical support and a potential framework for the accurate assessment of corneal biomechanical changes.

## 2. Materials and Methods

### 2.1. Construction of the Idealized FE Model with Air-Puff Loading

The dynamic deformation of the cornea during CVS measurement was simulated following a previously published procedure [25], except that an idealized corneal model with average human corneal dimensions was used in the current study. The model was constructed using self-developed software in the MATLAB programming environment (R2020a, MathWorks, Natick, MA, USA), which calls the finite element software Abaqus (version 2019, Dassault Systèmes Simulia Corp., Johnston, RI, USA) for actual FE analysis.

**Figure 1:**
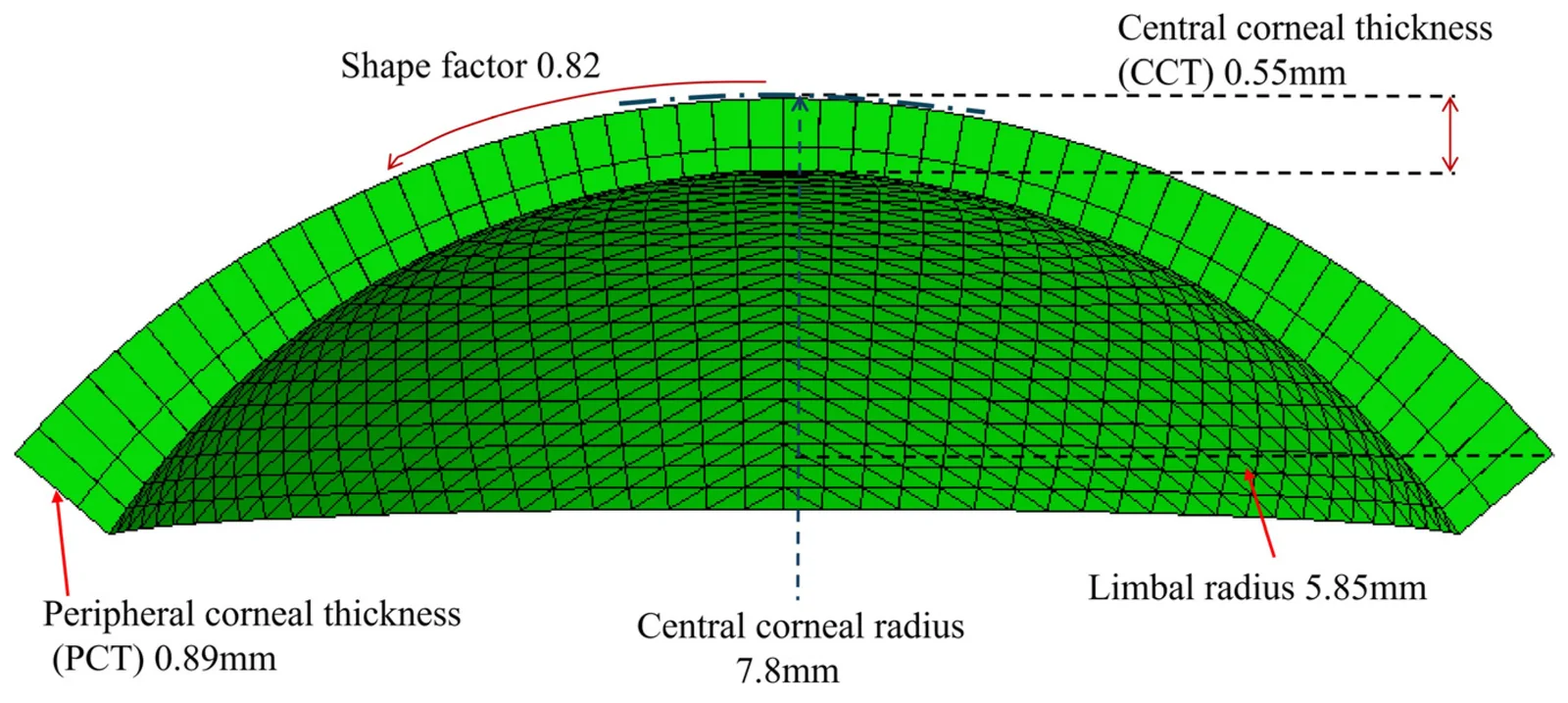
Basic structure of the idealized corneal model.

Briefly, the anterior corneal surface was modeled as an aspheric surface with a central radius of 7.8 mm and a shape factor of 0.82 (a value < 1 indicates a flatter shape toward the periphery), and the posterior surface was defined by an idealized prescribed thickness distribution increasing from 0.55 mm centrally to 0.89 mm toward the limbal region (Figure 1). This idealized thickness profile was introduced to approximate the physiological trend of peripheral thickening and provide geometric continuity for the FE model. The cornea was discretized with 15-node quadratic triangular prism elements (C3D15H) arranged in 24 rings and two elements through the thickness, yielding 4608 elements. The two numerical layers were assigned an anterior-to-posterior thickness ratio of 2:1 to represent the greater biomechanical contribution of the anterior stroma [26]. These idealized dimensions and the mesh topology followed the previously published CVS simulation workflow [25]. A roller support was imposed at the limbus [27], and the intraocular pressure (IOP) on the posterior corneal surface was set to 15 mmHg, the mean in healthy adults [28]. Because corneal properties depend on hydration and thickness [29], the fixed thickness profile was used to isolate regional stiffness effects, rather than to represent subject-specific pachymetry. The prescribed thickness distribution and IOP were held constant across all simulations to isolate the effect of regional material stiffness changes.

**Figure 2:**
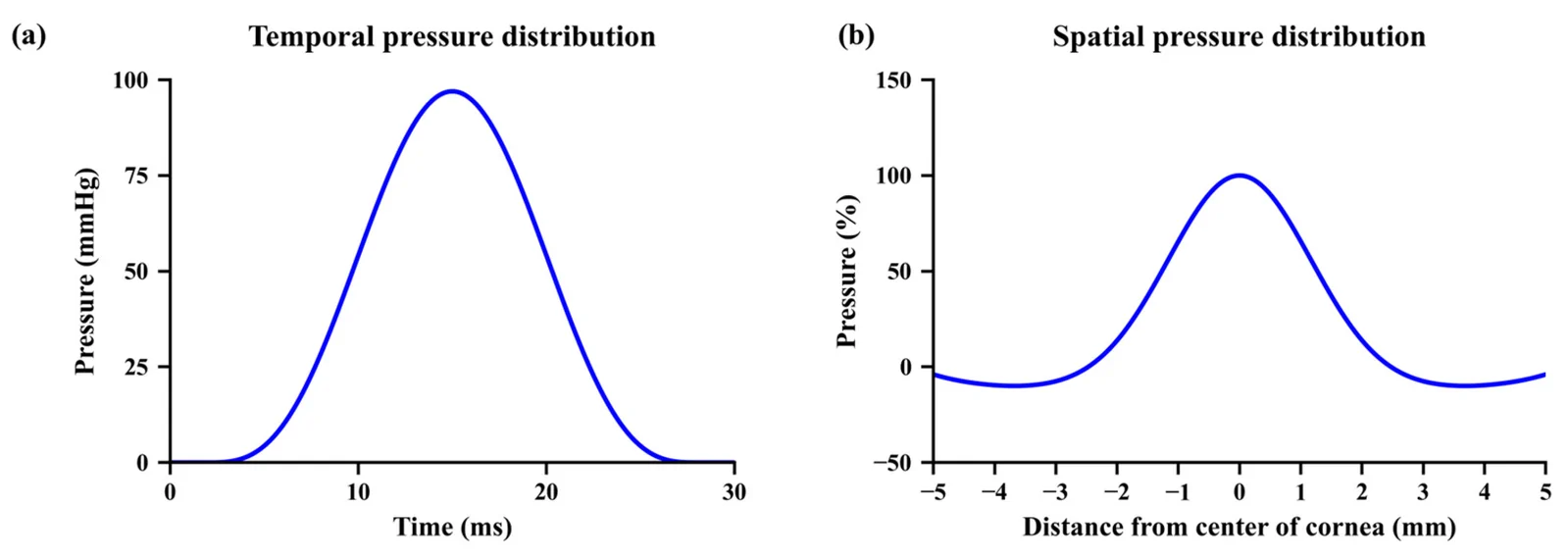
(**a**) Temporal variation in the air pressure at the middle of the air puff with a peak pressure of 98 mmHg [30], and (**b**) Spatial distribution of air-puff pressure across the corneal surface, expressed as a percentage of the peak pressure, adopted from a previous CFD study [31].

The air-puff loading was applied to the anterior corneal surface following the spatial distribution across the surface and the temporal variation in central air-puff pressure shown in Figure 2.

### 2.2. Regional Variations in Corneal Material Properties

In this study, the 1st-order Ogden material model was used to simulate the hyperelastic and nearly incompressible behavior of the cornea, which was found to accurately represent corneal material properties in a previous study [32]. The Ogden material formula used is $U=\frac{2\mu_{1}}{\alpha_{1}^{2}}\left({\bar{\lambda }}_{1}^{\alpha_{1}}+{\bar{\lambda }}_{2}^{\alpha_{1}}+{\bar{\lambda }}_{3}^{\alpha_{1}}-3\right)$, where U is the strain energy potential, ${\bar{\lambda }}_{i}$ are the main stretches, and $\mu_{1}$ and $\alpha_{1}$ represent the 1st order material parameters, which were set to 0.0485 and 108.57, respectively, based on previous ex vivo experiments of human corneal tissues [33,34].

**Figure 3:**
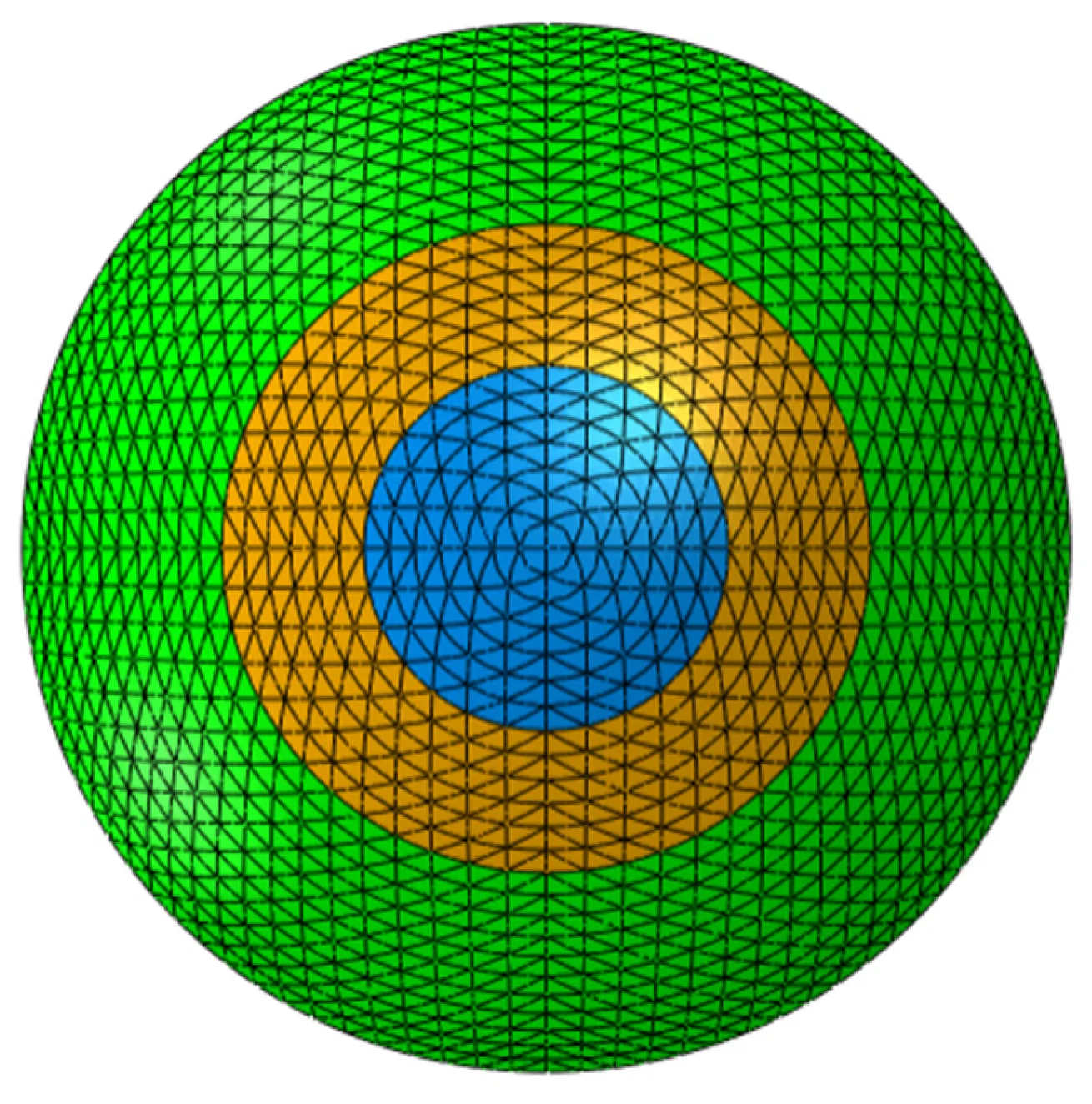
Schematic of regional corneal material property settings, with the central region with a radius ranging from 0 to 1.5 mm (blue region), the paracentral region from 1.5 to 3 mm (orange region), and the peripheral region from 3 to 5.8 mm (green region).

To evaluate the effects of regional material changes on biomechanical measurements, the cornea was divided into three concentric regions: central (radius 0–1.5 mm), paracentral (radius 1.5–3 mm), and peripheral (radius 3–5.8 mm), following a previous study [35] (Figure 3). Regional variations were implemented by adjusting the initial shear-modulus parameter mu1 within one region while holding the nonlinearity parameter alpha1 constant. Values from 70% to 130% of baseline, in 5% increments, represented regional softening and stiffening. These idealized scenarios were intended to approximate, rather than reproduce, spatial mechanical alterations that may occur in central keratoconus, after intracorneal ring segment implantation, or in peripheral corneal degeneration.

### 2.3. Calculation of CVS Parameters Based on Simulation Results

**Figure 4:**
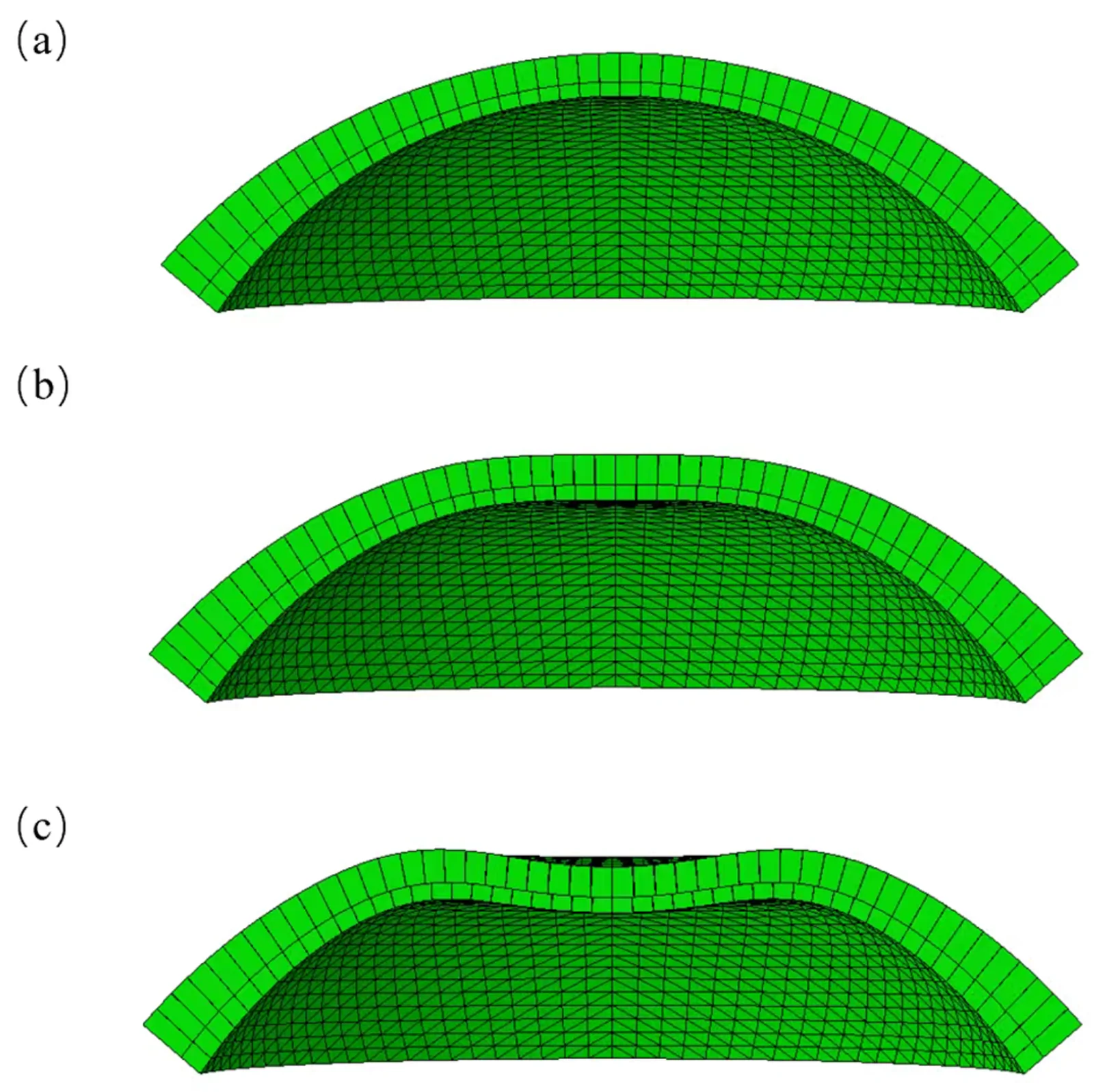
Cross-sectional view of the cornea during its dynamic deformation under air-puff loading: (**a**) initial state, (**b**) moment of first/second applanation, and (**c**) moment of highest concavity.

**Figure 5:**
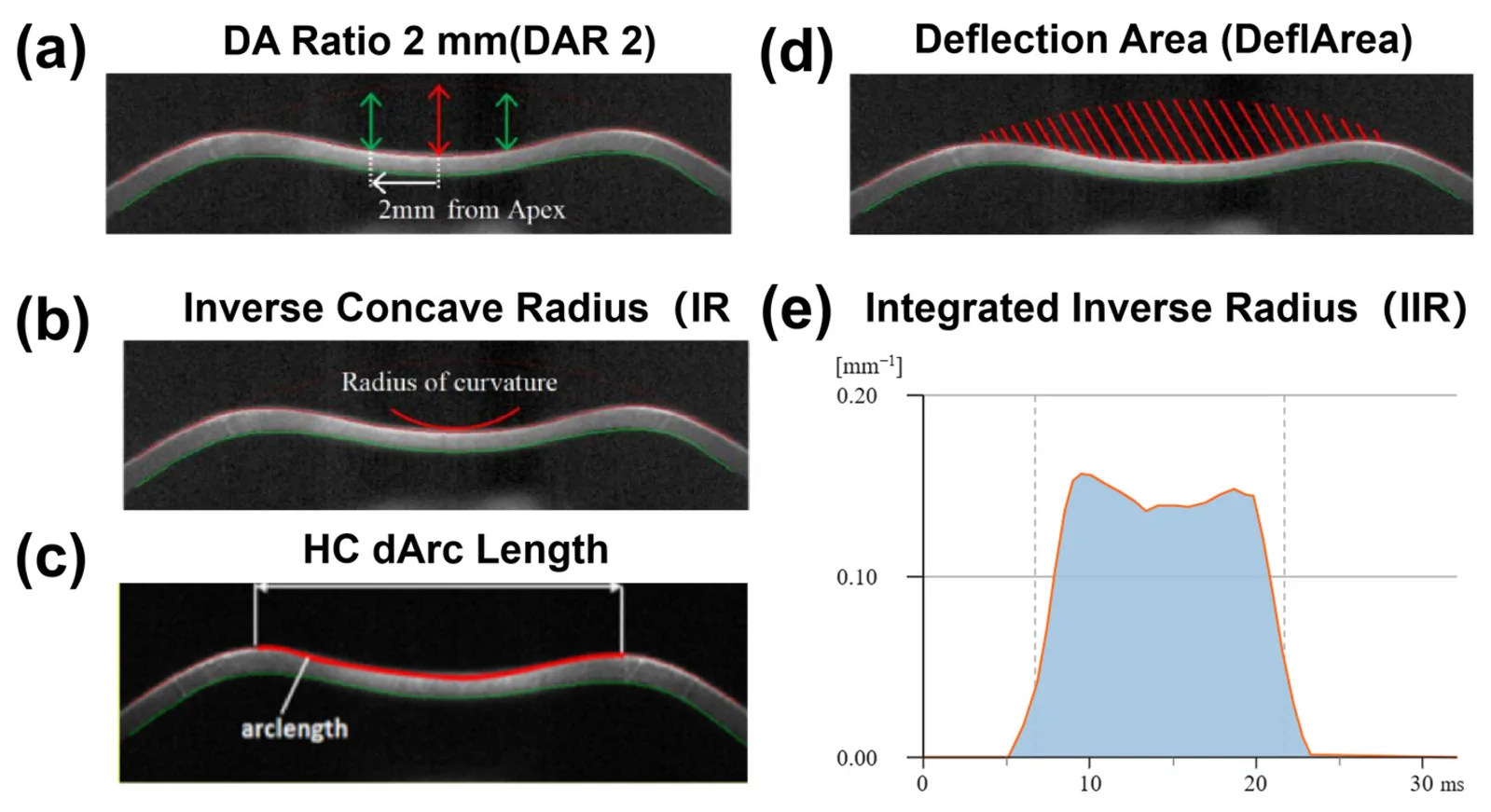
Schematic diagram of typical CVS parameters: (**a**) DA Ratio 2 mm, (**b**) IR, (**c**) HC dArc Length, (**d**) DeflArea, (**e**) IIR.

The nodal coordinates of the anterior corneal surface at each time step throughout the air-puff loading were extracted from the simulation results (Figure 4), and a set of biomechanical parameters was then calculated according to their definitions in CVS. A total of eight CVS parameters widely used in clinical biomechanical analysis (e.g., KC diagnosis and LVC evaluation) [36,37] were adopted in this study (Figure 5 and Table 1). The choice of this parameter set is also based on considerations of mathematical complexity and computational reliability. For example, composite parameters such as the Corvis Biomechanical Index (CBI) and the Tomographic Biomechanical Index (TBI) are frequently used in clinical studies; however, the explicit formulas are not publicly available.

### 2.4. Clinical Data for Model Assessment

To assess the clinical plausibility of the FE model, in vivo CVS measurements from 100 healthy subjects scheduled for corneal refractive surgery were analyzed. One eye per subject was included (100 eyes). Baseline characteristics are summarized in Table 2. Data were collected by a single experienced operator using CVS (software version 1.6r2187). Only examinations assigned an instrument quality rating of “OK” were retained, and the mean of three consecutive measurements was used for each eye. The available cohort was used for descriptive plausibility assessment; no inverse modeling, cohort-based calibration, or estimation of subject-specific material parameters was performed. The study complied with the Declaration of Helsinki and was approved by the Ethics Committee of the Eye Hospital of Wenzhou Medical University (No. 2023-032-K-25).

### 2.5. Statistical Analysis

A multi-index comparison approach was adopted to evaluate the clinical plausibility of the simulated results relative to clinical measurements from the normal healthy population described above, comprising three components: Z-score analysis, relative error (RE), and 95% reference interval (RI). The Z-score is calculated as *Z* = (*Y*_b_ − *μ*)/*σ*, where *Y*_b_ represents the value of a specific CVS parameter calculated from the simulation results of the baseline model (100% stiffness), and *μ* and *σ* denote the mean and standard deviation of the corresponding clinical measurements. The relative error is given as RE = |*Y*_b_ − *μ*|/|*μ*| × 100%, and the 95% RI is defined as *μ* ± 1.96*σ*.

Univariate linear regression analysis was employed to evaluate the responses of CVS parameters to changes in regional material stiffness. To enable comparison across biomechanical parameters with different units and numerical scales, all parameter values were normalized to their corresponding baseline values obtained from the baseline model. The relative parameter change was calculated as *Y*_rel_ = (*Y* − *Y*_b_)/|*Y*_b_| × 100%. The linear regression was then performed using *Y*_rel_ = *a* + *bX*, where *b* represents the percentage change in the CVS parameter per 1% change in material stiffness (*X*). The coefficient of determination (*R*^2^) and the *p*-value of the regression were included to assess the linearity and statistical significance of the response. *p*-value < 0.05 was considered statistically significant for all tests. The direction of the response was determined by the sign of *b*, whereas the sensitivity level was descriptively classified as low (|*b*| < 0.1), moderate (0.1 ≤ |*b*| < 0.5), high (0.5 ≤ |*b*| < 1.0), or very high (|*b*| ≥ 1.0).

All calculations were performed using Python (version 3.9; Python Software Foundation, Wilmington, DE, USA).

## 3. Results

### 3.1. Clinical Plausibility Assessment of Simulation Results

To evaluate clinical plausibility, the selected CVS parameters from the baseline simulation were compared descriptively with measurements from the 100 healthy eyes characterized in Table 2. All eight simulated values fell within the corresponding 95% reference intervals (Table 3), and relative errors ranged from 0.49% to 13.84%, with six values below 10%, demonstrating favorable consistency and close correspondence between simulated outcomes and clinical measurements [38]. These results indicate that the simulated data fell within a clinically reasonable range. This agreement suggests that the use of population-representative geometry, loading, and material inputs aligns well with the breadth of clinical reference intervals. No inverse modeling or fitting to this cohort was performed; consequently, the comparison supports plausibility but does not establish accuracy, uniqueness, or external validity.

### 3.2. Response Trends of CVS Parameters Against Regional Corneal Material Changes

The linear regression results are presented in Table 4. Within the simulated stiffness range, all parameters changed monotonically across the three regions. Coefficients of determination (R^2^) were close to 1.0 (all *p* values < 0.001), except for IIR in response to peripheral material changes (R^2^ = 0.440, *p* = 0.013). These regressions summarize the sampled model responses and should not be taken to demonstrate linear behavior outside the investigated range.

**Figure 6:**
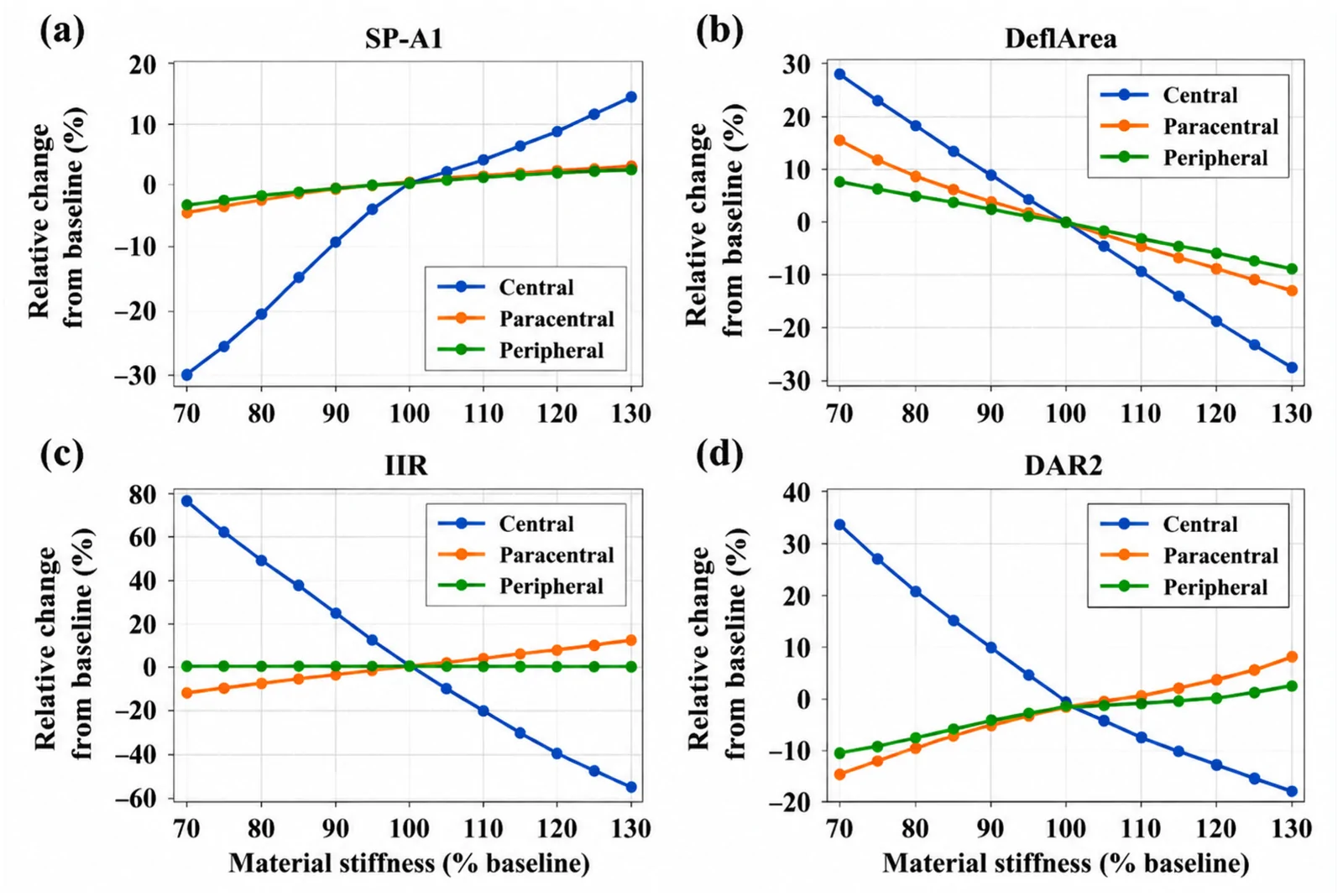
Normalized responses of CVS parameters to changes in corneal stiffness across different corneal regions. (**a**) SP-A1; (**b**) DeflArea; (**c**) IIR; (**d**) DAR2. Each subplot shows the percentage change relative to baseline as a function of material stiffness (% of baseline) for the central, paracentral, and peripheral corneal regions.

Sensitivity, categorized by the magnitude of slope *b*, revealed a pronounced gradient in the biomechanical parameters across corneal regions. Central corneal changes have the strongest overall influence, with most parameters showing high to very high sensitivity (|*b*| ≥ 0.5). For changes in the paracentral cornea, the parameter sensitivity became mostly moderate (0.1 ≤ |*b*| < 0.5), which further decreased to low (|*b*| < 0.1) to moderate in response to peripheral stiffness changes. It should be noted that the sensitivity of DAR1 remained low for all corneal regions. Furthermore, IIR and IR showed low sensitivity to changes in peripheral stiffness. Meanwhile, SP-A1 showed high sensitivity to central corneal changes but markedly reduced responses to both paracentral and peripheral corneal changes. These trends are better illustrated in Figure 6 and Supplementary Figure S1.

More importantly, divergent responses of the same biomechanical parameter to stiffness changes were observed across corneal regions. For example, DeflArea decreased with increasing stiffness (70% to 130%) across all corneal regions, whereas DAR2 decreased with material stiffening in the central region but reversed to an increasing trend with paracentral or peripheral stiffening. These behaviors introduced ambiguities in the clinical interpretation of CVS parameter values and their changes. As shown in Table 5 (Case 1), a 5% increase in central corneal stiffness and a 30% reduction in paracentral stiffness can result in similar IIR values (7.65 vs. 7.64), and the same reduction in paracentral stiffness can replicate the DAR2 value of a 25% increase in the central region (3.54 vs. 3.52). Similarly, it is not clear whether an increase in either IIR or DAR2 is caused by corneal softening in the central region or stiffening in the paracentral region (Case 2).

### 3.3. Parameter Combination to Map Regional Material Changes

The divergent trends in CVS parameters in response to regional changes in corneal material, in turn, offered an opportunity to map those changes using the combined behavior of multiple parameters. However, the current parameters remain insufficient to independently distinguish among the variations in the three regions, because their response behaviors only exhibit two distinct patterns.

**Figure 7:**
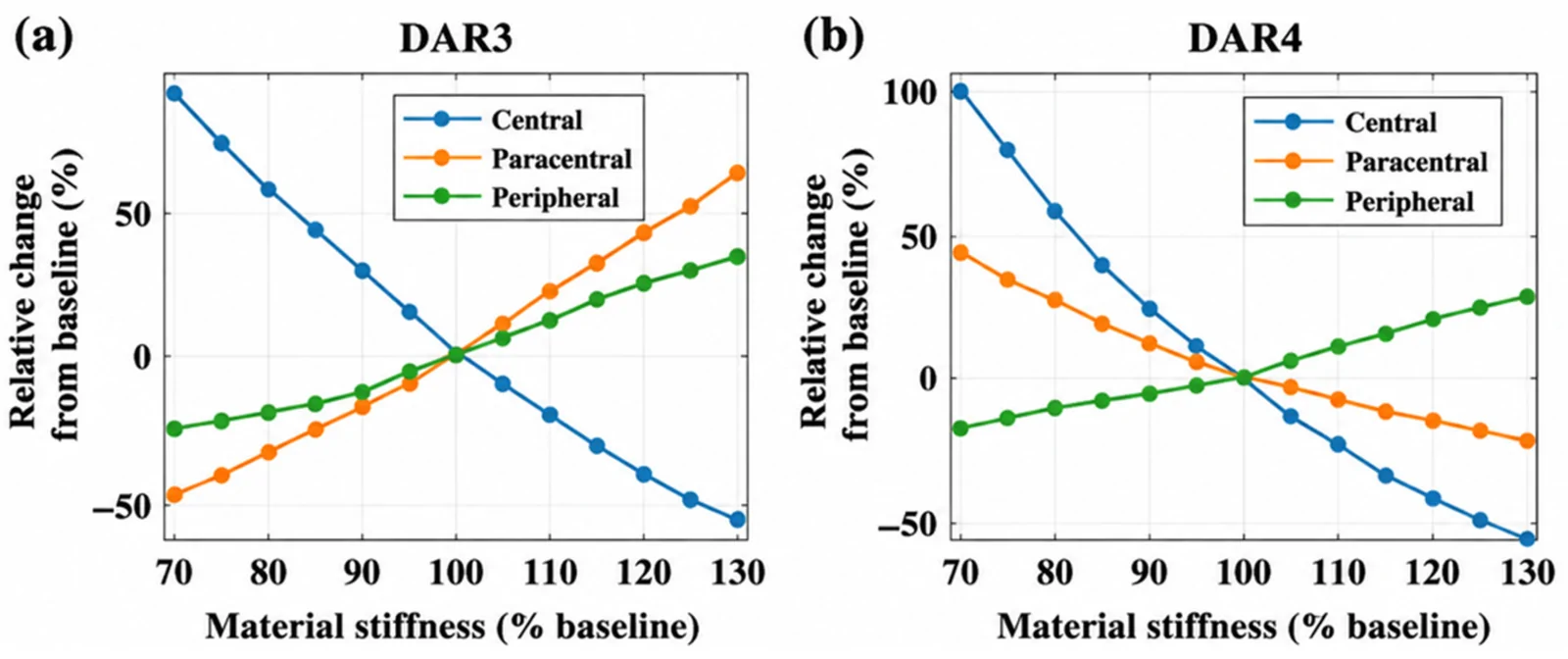
Normalized responses of DAR3 and DAR4 to changes in corneal stiffness across different corneal regions, showing reversed trends in response to paracentral stiffness changes. (**a**) DAR3; (**b**) DAR4. Each subplot shows the percentage change relative to baseline as a function of material stiffness (% of baseline) for the central, paracentral, and peripheral corneal regions.

This can be remedied by introducing a series of DA Ratio X mm parameters that account for corneal deformation over a wider range than the already-analyzed DAR1 and DAR2. Three cases, namely 3 mm, 4 mm, and 5 mm, were explored in this study, and the calculations followed the definitions of DAR1 and DAR2 in Table 1. As shown in Table 6 and Figure 7, while DA Ratio 3 mm (DAR3) shared the same pattern as DAR1 and DAR2, DAR4 and DAR5 showed a reversed trend for paracentral stiffness change, thereby introducing a third response pattern.

Table 7 collectively shows the three patterns. Specifically, parameters in Pattern A decrease or increase uniformly with stiffness change across all regions; those in Pattern B decrease when stiffness increases in the central and paracentral regions and increase when that happens in the peripheral region; finally, parameters in Pattern C decrease when stiffness increases from 70% to 130% in the central region and increase when that happens in other regions.

**Figure 8:**
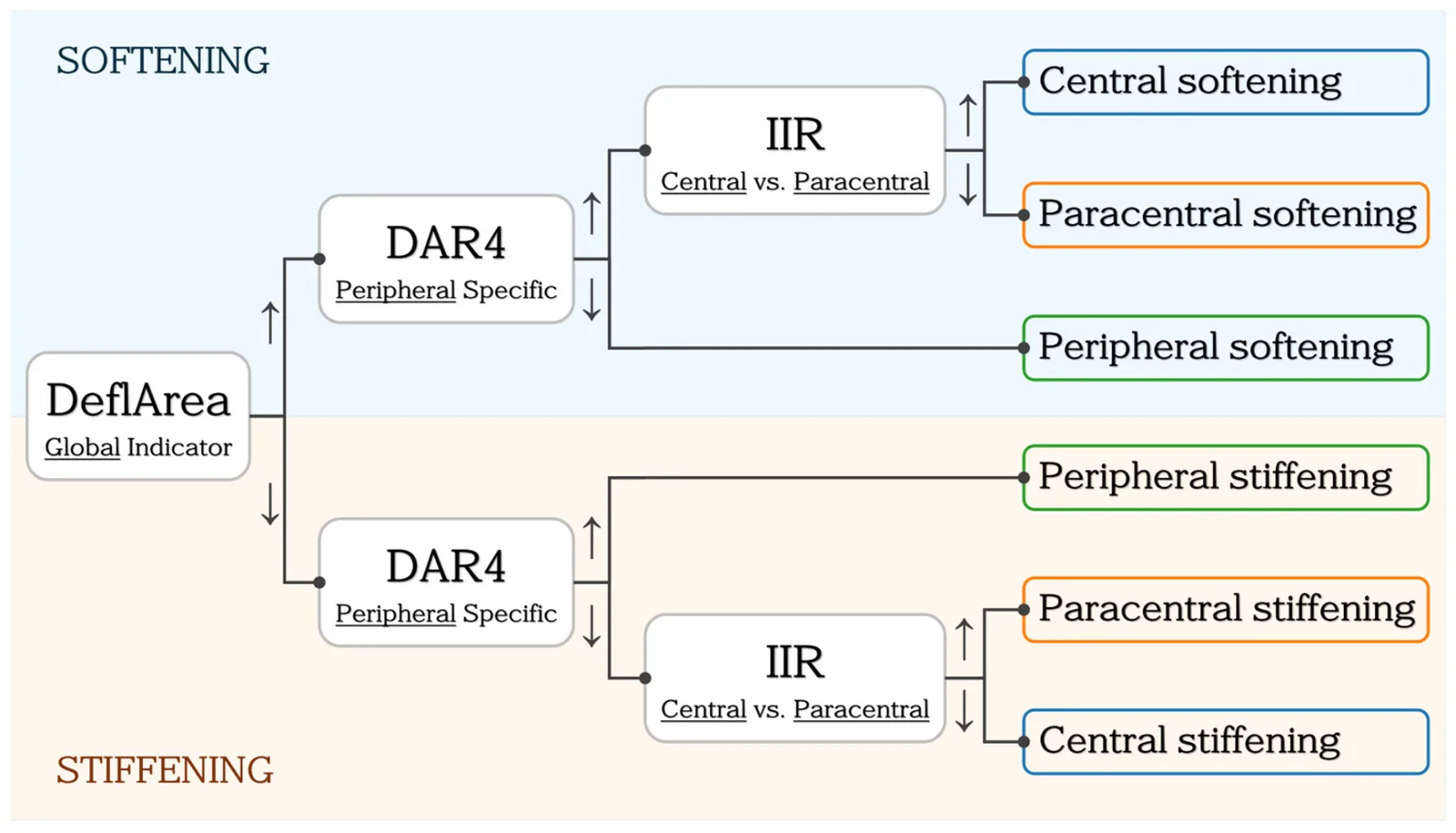
Mapping regional corneal stiffness changes using the parameter combination of DeflArea, DAR4, and IIR.

Therefore, selecting one parameter from each pattern enables mapping regional material changes. By balancing parameter sensitivity, clinical familiarity, and computational complexity, a parameter combination of DeflArea, DAR4, and IIR was selected. In practice, as shown in Figure 8, DeflArea indicates global softening or stiffening across all corneal regions, DAR4 classifies the peripheral region, and IIR distinguishes central and paracentral stiffness changes.

## 4. Discussion

CVS parameters are core clinical indicators for evaluating global corneal biomechanics. Numerical simulations revealed that IIR and DAR2 rose with central corneal softening, which is consistent with the elevated trends after refractive surgery [19]. In contrast, both parameters declined with peripheral corneal softening. This highlights that parameter changes depend on both the nature of material alterations and their spatial location, with identical mechanical changes inducing opposite parameter trends in different corneal regions. This indicates that certain CVS parameters exhibit significant regional dependence, and their alterations do not directly correspond to global corneal stiffness changes.

Beyond regional dependence, this study also revealed the interpretive non-uniqueness inherent in single-parameter assessment. Simulations demonstrated that mechanically opposing alterations in different corneal regions can yield similar parameter trends or values. Most notably, central softening, a hallmark of post-LVC wound healing, and paracentral stiffening, characteristic of ICRS implantation [39], could produce indistinguishable trends in IIR and DAR2 measurements despite representing diametrically opposed pathophysiological processes. These findings indicate that similar parameter values or changing trends may mask fundamentally different underlying mechanical conditions and may be insufficient to infer regional material alterations. Furthermore, this interpretive non-uniqueness may partly explain why subclinical keratoconus, which typically begins with localized paracentral biomechanical weakening, can remain undetected by conventional CVS [40,41].

Although material property alterations in different corneal anatomical regions may induce similar parameter fluctuations, the biomechanical response patterns underlying identical parameter change trends exhibited significant heterogeneity across regions. Quantitative simulation results demonstrated that to elicit an equivalent degree of biomechanical parameter change, the peripheral corneal material stiffness required a reduction of approximately 30%, whereas the central cornea required less than a 10% reduction in material property to achieve the same effect. Aggregation of multiple simulation datasets suggested that alterations in central corneal material properties consistently produced more pronounced parameter changes. This indicates that single global parameters cannot characterize intervention-related biomechanical changes or reflect the actual central corneal biomechanical status, especially following CXL and ICRS implantation [42,43]. Consequently, parameter interpretation in clinical practice may need to account for this inherent regional sensitivity gradient to avoid underestimating the significance of peripheral changes or overinterpreting the implications of central parameter shifts, even though direct clinical relevance has yet to be established.

The present study further revealed the inherent potential of the DA Ratio X mm parameter set in characterizing regional variations in corneal material properties. When the material stiffness of the central cornea was reduced, simulation results demonstrated that all DA Ratio X mm parameter values exhibited a significant upward trend (i.e., a negative response). Whereas a consistent decrease in all DA Ratio X mm parameter values was observed in simulations when the material stiffness of the peripheral region was decreased (i.e., a positive response). In contrast, a distinct spatial heterogeneity in the influence pattern was observed when the material stiffness of the paracentral region was reduced. This spatially heterogeneous pattern, characterized by a positive response in DAR1-DAR3 and a negative response in DAR4 and DAR5, may serve as a characteristic fingerprint for helping distinguish paracentral corneal softening.

The findings described above collectively underscore the inadequacy of single-parameter assessment and motivated a systematic analysis of parameter response characteristics in this study. By quantifying the response direction of each parameter to material property alterations across different corneal regions and performing hierarchical classification analysis, all evaluated parameters were categorized into three distinct pattern groups. Pattern A (SP-A1, DeflArea, DeflA Max, HCdArcLength) responded uniformly across all regions, reflecting global stiffness changes without regional differentiation; Pattern B (DAR4, DAR5) demonstrated partial cross-regional discriminative capability that distinguishes the peripheral region; and Pattern C (IIR, IR, DAR1–DAR3) responded differently between the central and paracentral regions. Crucially, no single parameter within any cluster was capable of uniquely distinguishing all six types of regional material alteration considered, thereby establishing the necessity of a multi-parameter combination approach for regional biomechanical assessment.

Based on these findings, this study screened and selected three corneal biomechanical parameters (DeflArea, DAR4, IIR) to infer potential regional mechanical abnormalities from their combined changes. Within this simulation framework, distinct response patterns can be used to distinguish regional corneal mechanical alterations, e.g., (1) synchronous increases in DeflArea, DAR4, and IIR suggest central corneal softening; (2) synchronous increases in DeflArea and DAR4 with decreased IIR point to predominant paracentral weakening; (3) whereas increased DeflArea with decreased DAR4 (regardless of IIR trend) indicates peripheral corneal material softening. This finding suggests that regional alterations in corneal material properties may be distinguishable via multi-parameter combinations, pointing to a potential novel method for regional identification and biomechanical assessment of the cornea. Regional biomechanical assessment may provide a theoretical basis for investigating focal ectatic weakening and biomechanical changes after refractive surgery. It may also help evaluate spatial effects of treatments such as corneal cross-linking and intracorneal ring segments. The present results identify testable hypotheses for these settings but do not demonstrate improved disease detection or treatment monitoring.

Several limitations constrain interpretation and define priorities for future work. First, the current model used idealized concentric regions, homogeneous regional properties, fixed IOP and thickness distribution, and simplified limbal support. Future studies should systematically vary IOP and CCT to quantify their interactions with regional stiffness changes and incorporate patient-specific models based on measured pachymetry and realistic lesion geometries. Second, the isotropic Ogden formulation and simplified through-thickness discretization did not account for collagen fiber anisotropy, hydration-dependent behavior, and continuous stromal gradients [29,44,45,46], and mesh convergence was not formally assessed. Incorporating fiber- and depth-dependent material properties could alter both the magnitude and spatial distribution of air puff-induced deformation. Although the qualitative regional dependence observed here may persist, its quantitative expression, including the response slopes, relative sensitivity rankings, and potentially the assignment of some parameters to the three response patterns, could differ under such formulations. Future studies should therefore evaluate fiber-reinforced formulations, improve through-thickness discretization with formal convergence testing, and conduct uncertainty analyses to determine whether increased model complexity improves predictive reliability [23,24]. Third, although detailed reporting of model inputs and procedures facilitates computational reproducibility, the proposed parameter combination has not yet been independently replicated or validated in patient-specific models or clinical datasets. Future investigations should establish predefined analysis protocols with appropriate sample-size estimation and perform independent validation using prospectively collected multicenter cohorts. Moreover, advances in ophthalmic optical imaging have demonstrated increasing capabilities for spatially resolved ocular measurements, including extended-range swept-source acquisition, directional OCT, and automated wide-field imaging [47,48,49]. These developments may provide future opportunities for integrating high-resolution imaging with biomechanical modeling of anterior segment tissues; however, their application to corneal biomechanics requires dedicated validation.

## 5. Conclusions

In this idealized finite element study, CVS parameter responses depended on the prescribed location of regional material changes, and similar single-parameter values could arise from different modeled conditions. The combination of DeflArea, DAR4, and IIR demonstrated distinct response patterns among the modeled central, paracentral, and peripheral scenarios, suggesting its potential value for future regional biomechanical assessment. Further investigations incorporating patient-specific geometries, experimental measurements, and clinical datasets are warranted to evaluate the applicability of this framework in physiological settings.

## Figures and Tables

**Table 1:** Definition of corneal biomechanical parameters adopted in this study.

Parameter	Definition	Timing	Current Interpretation
Inverse Concave Radius (IR)	Inverse radius of the curvature at the corneal apex at maximum concavity	HC	Smaller radius (larger inverse radius) indicates weaker corneal resistance to deformation
Integrated Inverse Radius (IIR)	Concave curvature integrated between first and second applanation	Between A1 and A2	Higher values indicate greater overall corneal deformability and reduced stiffness
DA Ratio 1 mm (DAR1)	Ratio between the deformation amplitude at the apex and that 1 mm away	Close to A1	Higher ratio indicates reduced stiffness
DA Ratio 2 mm (DAR2)	Ratio between the deformation amplitude at the apex and that 2 mm away	Close to A1	Higher ratio indicates greater central–paracentral deformation asymmetry and reduced stiffness
HC dArc Length (HCdArcLength)	Arc length change from the initial state to the highest concavity	HC	Usually negative, larger change (absolute value) indicates increased stiffness
SP-A1	Stiffness parameter at first applanation	A1	Higher values indicate greater corneal rigidity and stronger resistance to deformation
Deflection Area (DeflArea)	Area between the initial contour line and that at highest concavity	HC	Larger area indicates greater overall corneal deformability and reduced stiffness
DeflectionAmpMax (DeflAMax)	Maximum inward displacement of the corneal apex	HC	Larger displacement indicates greater overall corneal deformability and reduced stiffness

A1: first applanation, HC: highest concavity, A2: second applanation.

**Table 2:** Baseline characteristics of the 100 subjects.

Parameters	Values
Age (years)	22 (18, 26)
CCT (μm)	542.35 ± 22.63
bIOP (mm Hg)	16.07 ± 2.43

Data are presented as mean ± SD except for age, which is expressed as median (IQR) due to non-normal distribution.

**Table 3:** Comparison of simulated data with clinical measurements.

Parameter	Simulated	Clinical (Mean ± SD)	Z-Score	RE (%)	Within 95% RI
IIR	8.58	7.73 ± 0.91	0.93	11.00	Yes
IR	0.15	0.16 ± 0.03	−0.33	6.25	Yes
DAR1	1.37	1.59 ± 0.22	−1.00	13.84	Yes
DAR2	4.06	4.08 ± 0.38	−0.05	0.49	Yes
DeflArea	2.87	2.93 ± 0.43	−0.14	2.05	Yes
DeflAMax	1.40	1.36 ± 0.28	0.14	2.94	Yes
HCdArcLength	−0.14	−0.15 ± 0.22	0.05	6.67	Yes
SP-A1	113.62	109.71 ± 16.01	0.24	3.56	Yes

SD: standard deviation, RE: relative error (%), RI: reference interval.

**Table 4:** Linear regression analysis of percentage changes in CVS parameters versus regional corneal material alteration.

Parameter	Region	*b*	*R* ^2^	*p*-Value	Sensitivity
IIR	Central	−2.282	0.988	<0.001	Very high
	Paracentral	0.427	0.994	<0.001	Moderate
	Peripheral	0.019	0.440	0.013	Low
IR	Central	−2.049	0.992	<0.001	Very high
	Paracentral	0.314	0.999	<0.001	Moderate
	Peripheral	−0.019	0.971	<0.001	Low
DAR1	Central	−0.093	0.993	<0.001	Low
	Paracentral	0.087	0.998	<0.001	Low
	Peripheral	0.027	0.962	<0.001	Low
DAR2	Central	−0.758	0.962	<0.001	High
	Paracentral	0.350	0.988	<0.001	Moderate
	Peripheral	0.238	0.960	<0.001	Moderate
DeflArea	Central	−0.812	0.995	<0.001	High
	Paracentral	−0.430	0.987	<0.001	Moderate
	Peripheral	−0.268	0.999	<0.001	Moderate
DeflAMax	Central	−0.810	0.996	<0.001	High
	Paracentral	−0.269	0.986	<0.001	Moderate
	Peripheral	−0.190	0.999	<0.001	Moderate
HCdArcLength	Central	−2.743	0.996	<0.001	Very high
	Paracentral	−0.837	0.979	<0.001	High
	Peripheral	−0.272	0.992	<0.001	Moderate
SP-A1	Central	0.612	0.960	<0.001	High
	Paracentral	0.087	0.954	<0.001	Low
	Peripheral	0.078	0.952	<0.001	Low

**Table 5:** Similar values or same changing trends of IIR and DAR2 in response to stiffness alteration across different corneal regions.

Case	Material Stiffness (%)	Region	IIR	DAR2	Observation
1	105	Central	7.65	3.93	Similar values
	70	Paracentral	7.64	3.54	
	125	Central	4.39	3.52	
2	100	ALL	8.58	4.06	Same trends
	130	Paracentral	9.80	4.44	
	70	Central	15.64	5.35	

**Table 6:** Linear regression analysis of percentage changes in DA Ratio X mm parameters versus regional corneal material alteration.

DA Ratio	Region	*b*	*R* ^2^	*p*-Value	Sensitivity
3 mm	Central	−2.282	0.988	<0.001	Very high
	Paracentral	1.670	0.990	<0.001	Very high
	Peripheral	1.046	0.985	<0.001	Very high
4 mm	Central	−2.675	0.963	<0.001	Very high
	Paracentral	−1.104	0.975	<0.001	Very high
	Peripheral	0.815	0.986	<0.001	High
5 mm	Central	−3.332	0.941	<0.001	Very high
	Paracentral	−1.462	0.975	<0.001	Very high
	Peripheral	0.436	0.999	<0.001	Moderate

**Table 7:** Response patterns of corneal biomechanical parameters to regional material alterations of the cornea, revealed by response slope *b*.

Pattern	Parameter	Central	Paracentral	Peripheral
A	DeflAMax	−0.810	−0.269	−0.190
	DeflArea	−0.812	−0.430	−0.268
	HCdArcLength	−2.743	−0.837	−0.272
	SP-A1	0.612	0.087	0.078
B	DAR4	−2.675	−1.104	0.815
	DAR5	−3.332	−1.462	0.436
C	IIR	−2.282	0.427	0.019
	IR	−2.049	0.314	−0.019
	DAR1	−0.093	0.087	0.027
	DAR2	−0.758	0.350	0.238
	DAR3	−2.282	1.670	1.046

Red colors indicate decreases in the parameter when material stiffness increases from 70% to 130%, green colors indicate increases, and darker colors reflect larger changes.

## Data Availability

The raw data supporting the conclusions of this article will be made available by the authors on request.

## Supplementary Materials

The following supporting information can be downloaded at: https://www.mdpi.com/article/10.3390/bioengineering13080904/s1, Figure S1: Normalized responses of CVS parameters to changes in corneal stiffness across different corneal regions.
