# Supplementary material for: Regional Dependence of Corvis ST Biomechanical Parameters on Corneal Material Properties: A Finite Element Study
Source: Bioengineering (Basel). 2026 Aug 10;13(8):904. doi: 10.3390/bioengineering13080904 (PMC13509431; doi:10.3390/bioengineering13080904)
Supplement: Supplementary file 1 [file bioengineering-13-00904-s001.zip › bioengineering-4429518-supplementary.pdf]

Supplemental Figure

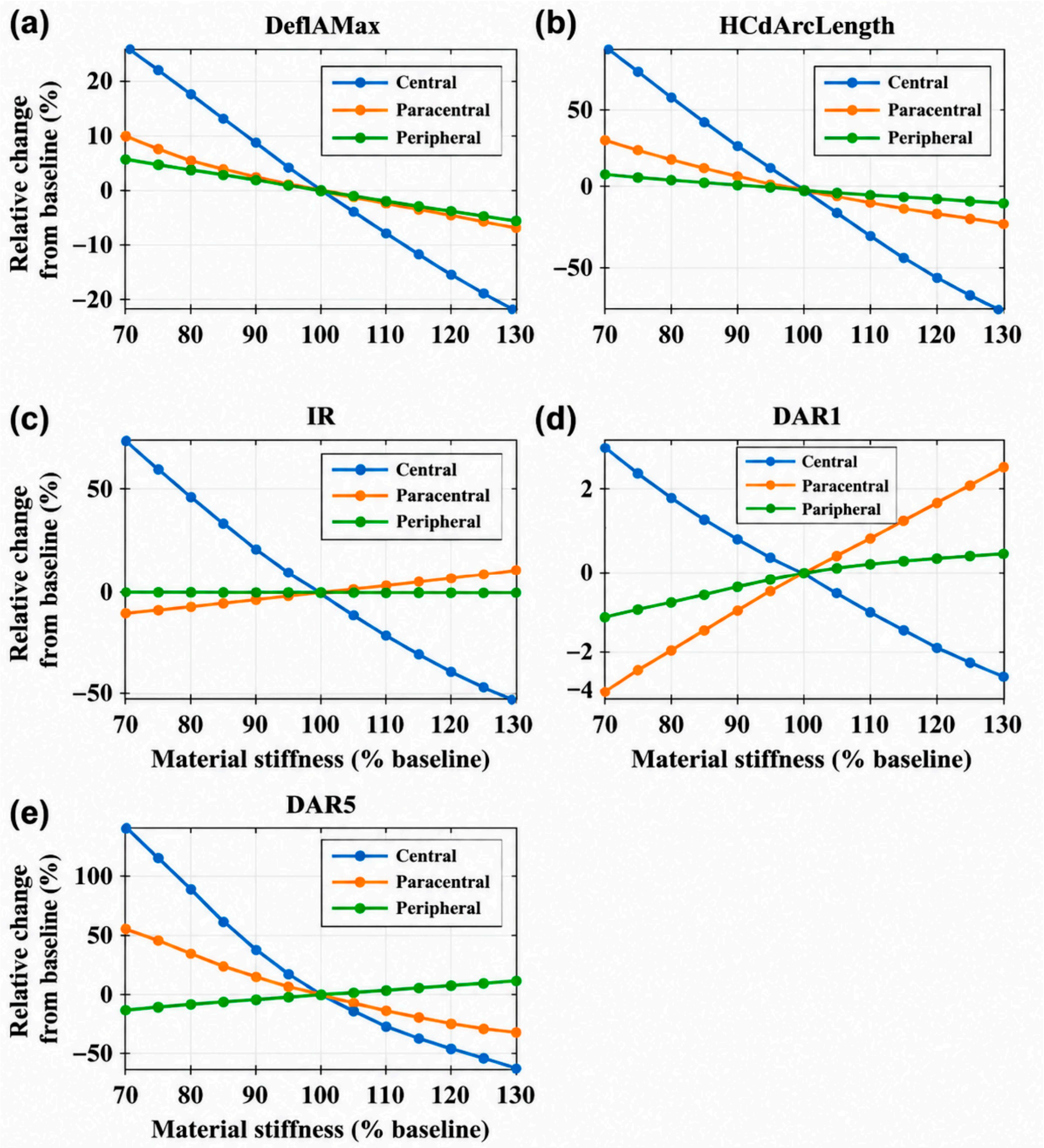

Figure S1. Normalized responses of CVS parameters to changes in corneal stiffness across different corneal regions. (**a**) DeflAmax; (**b**) HCdArcLength; (**c**) IR; (**d**) DAR1; (**e**)DAR5. Each subplot shows the percentage change relative to baseline as a function of material stiffness (% of baseline) for the central, paracentral, and peripheral corneal regions.
